# Analysis of EF-Hand Proteins in Soybean Genome Suggests Their Potential Roles in Environmental and Nutritional Stress Signaling

**DOI:** 10.3389/fpls.2017.00877

**Published:** 2017-05-24

**Authors:** Houqing Zeng, Yaxian Zhang, Xiajun Zhang, Erxu Pi, Yiyong Zhu

**Affiliations:** ^1^College of Life and Environmental Sciences, Hangzhou Normal UniversityHangzhou, China; ^2^College of Resources and Environmental Sciences, Nanjing Agricultural UniversityNanjing, China

**Keywords:** soybean (*Glycine max*), EF-hand motif, calcium signal, calmodulin, calmodulin-like protein (CML), calcineurin B-like protein (CBL), calcium-dependent protein kinase (CDPK), Rboh (respiratory burst oxidase homolog)

## Abstract

Calcium ion (Ca^2+^) is a universal second messenger that plays a critical role in plant responses to diverse physiological and environmental stimuli. The stimulus-specific signals are perceived and decoded by a series of Ca^2+^ binding proteins serving as Ca^2+^ sensors. The majority of Ca^2+^ sensors possess the EF-hand motif, a helix-loop-helix structure which forms a turn-loop structure. Although EF-hand proteins in model plant such as Arabidopsis have been well described, the identification, classification, and the physiological functions of EF-hand-containing proteins from soybean are not systemically reported. In this study, a total of at least 262 genes possibly encoding proteins containing one to six EF-hand motifs were identified in soybean genome. These genes include 6 calmodulins (CaMs), 144 calmodulin-like proteins (CMLs), 15 calcineurin B-like proteins, 50 calcium-dependent protein kinases (CDPKs), 13 CDPK-related protein kinases, 2 Ca^2+^- and CaM-dependent protein kinases, 17 respiratory burst oxidase homologs, and 15 unclassified EF-hand proteins. Most of these genes (87.8%) contain at least one kind of hormonal signaling- and/or stress response-related *cis*-elements in their -1500 bp promoter regions. Expression analyses by exploring the published microarray and Illumina transcriptome sequencing data revealed that the expression of these EF-hand genes were widely detected in different organs of soybean, and nearly half of the total EF-hand genes were responsive to various environmental or nutritional stresses. Quantitative RT-PCR was used to confirm their responsiveness to several stress treatments. To confirm the Ca^2+^-binding ability of these EF-hand proteins, four CMLs (CML1, CML13, CML39, and CML95) were randomly selected for SDS–PAGE mobility-shift assay in the presence and absence of Ca^2+^. Results showed that all of them have the ability to bind Ca^2+^. This study provided the first comprehensive analyses of genes encoding for EF-hand proteins in soybean. Information on the classification, phylogenetic relationships and expression profiles of soybean EF-hand genes in different tissues and under various environmental and nutritional stresses will be helpful for identifying candidates with potential roles in Ca^2+^ signal-mediated physiological processes including growth and development, plant-microbe interactions and responses to biotic and abiotic stresses.

## Introduction

During their life cycles, plants encounter a variety of external stresses such as heat, cold, drought, flooding, salinity, nutrient deficiency, and attacks from insects and pathogens. Calcium (Ca^2+^) is one of the most abundant elements on earth, and serves as a universal second intracellular messenger that plays a critical role in plant responses to environmental stresses, as well as hormonal and developmental cues (Sanders et al., 2002). Ca^2+^ concentration in the cytoplasm and nucleus is kept low (∼100–200 nM), since high levels of cytosolic Ca^2+^ are toxic to cells by forming insoluble compounds with phosphate derivatives and complex with macromolecules. A steep Ca^2+^ gradient across the plasma membrane as well as the inner membrane system enclosing the cellular Ca^2+^ storages is established under the controls of stimulus responsive Ca^2+^ channels, pumps and transporters (McAinsh and Pittman, 2009; Kudla et al., 2010). It has been well-documented that various external biotic or abiotic stimuli can quickly trigger specific and distinct spatial-temporal patterns of changes in cytosolic Ca^2+^ concentration, and “Ca^2+^ signatures" have been coined to describe the specificities of calcium signals triggered by the causative stimuli (Webb et al., 1996; McAinsh and Pittman, 2009). The stimulus-specific signals are perceived and decoded by a series of Ca^2+^ binding proteins which function as Ca^2+^ sensors to generate specific or overlapping responses (DeFalco et al., 2010; Batistic and Kudla, 2012). Upon Ca^2+^ binding, Ca^2+^ sensors undergo conformational changes that either promote their interactions with target proteins or alter their own enzymatic activities, and therefore transmit these stimulus-specific signals into biochemical, molecular, cellular or physiological events, which help plants survive the unfavorable environmental conditions.

The majority of Ca^2+^ sensors possess the EF-hand motif, which is composed of a 29-amino acid helix-loop-helix structure, with the central 12 residues forming a turn-loop structure that is responsible for the coordination of Ca^2+^ ion (Gifford et al., 2007). Within the coordination loop, ligating residues are found at positions 1, 3, 5, 7, 9, and 12 (X^∗^Y^∗^Z^∗^-Y^∗^-X^∗∗^-Z, “^∗^” represents an intervening residue). The loop is enriched in negatively charged residues, and a Gly at position six allows the loop to wrap around the Ca^2+^ ion, a feature critical for high-affinity Ca^2+^ binding (Gifford et al., 2007). Structural properties of the EF-hand motif allow for rapid on/off Ca^2+^ binding, which permits quick responses to changes in cytosolic Ca^2+^ concentration. Typically, EF-hands occur in pairs and facilitate high-affinity cooperative binding of Ca^2+^ (DeFalco et al., 2010). Calmodulins (CaMs) and CaM-like proteins (CMLs), calcineurin B-like proteins (CBLs), and calcium-dependent protein kinases (CDPKs) are the three largest groups of Ca^2+^ sensors in plants (Luan et al., 2002; DeFalco et al., 2010; Zeng et al., 2015). CaMs are ubiquitous Ca^2+^-binding proteins existing in all eukaryotes, whereas CMLs, CDPKs, and CBLs are restricted to plant taxa and certain protist groups (Poovaiah et al., 2013; Hamel et al., 2014; Kleist et al., 2014; Zhu et al., 2015). In *Arabidopsis thaliana* genome, there are at least 250 genes encoding for EF-hand proteins which include 7 CaM genes, 50 CML genes, 10 CBL genes, 34 CDPK genes (Day et al., 2002; Hrabak et al., 2003; McCormack and Braam, 2003; Kolukisaoglu et al., 2004). However, the identification, classification and the physiological functions of EF-hand-containing proteins in other plants are largely unaddressed.

Soybean is an important economic crop which provides an oil- and protein-rich food for human around the world, and also an important crop for the production of renewable fuel of biodiesel. However, global productivity of soybean is frequently affected by various environmental stresses, such as drought, salt, and nutrient deficiency (Tran and Mochida, 2010; Zeng et al., 2010; Deshmukh et al., 2014). With the availability of whole-genome sequences of soybean, genes encoding proteins with specific domains like EF-hand motif can be identified at the whole-genome scale (Schmutz et al., 2010). In this study, we carried out a genome-wide search and identified and analyzed all the available EF-hand motif-containing proteins in soybean genome.

## Materials and Methods

### Identification of EF-Hand Proteins from Soybean

Proteins containing an EF-hand domain or in the family of Ca^2+^-binding proteins which included domains PF00036, PTHR10891, PS00018, PS50222, SM00054, and SSF47473, were collected by keyword searching from the Phytozome’s soybean database^1^. Furthermore, “EF-hand,” “calmodulin,” “calmodulin-like,” “calcineurin B-like,” and “calcium-dependent protein kinase” were each used as a key word to perform homolog searches. In addition, the amino acid sequences of Arabidopsis CaM2, CBL1, CDPK1, CRK1, and RbohD were used as queries to blast against the soybean genome database using the BlastP program. The nucleotide and protein sequences were obtained and analyzed for EF-hands and other domains using InterPro database^2^ (Hunter et al., 2009). Proteins not showing EF-hand domains were eliminated from the list.

### Phylogenetic Analysis and Chromosomal Mapping

Sequences of EF-hand proteins of Arabidopsis and soybean were obtained from TAIR^3^ and Phytozome^4^, respectively. The amino acid sequences of EF-hand proteins were aligned using ClustalW, and a phylogenetic tree was constructed by the neighbor-joining method using the software MEGA6 (Tamura et al., 2013). The location of soybean genes encoding EF-hand proteins was determined based on their physical positions on chromosomes corresponding to their locus number in the JBrowse genome browser^5^. If the distance between two neighboring paralogous genes were less than 100 kb and separated by five or less genes, they were considered to be tandemly duplicated genes (Zhu et al., 2016).

### Identification of Conserved Domains and *cis*-Acting Regulatory Elements

Conserved domains other than EF-hand motif were predicted using InterPro^6^. Myristoylation sites were predicted using PlantsP^7^. Promoter sequences of 1.5 kb upstream to the transcription start site of the genes encoding EF-hand proteins were retrieved from JBrowse, and the location of stress-related *cis*-acting regulatory elements was analyzed using Regulatory Sequence Analysis Tools^8^ (Medina-Rivera et al., 2015).

### Microarray and RNA-Seq Datasets

Genome-wide public RNA-seq datasets [Reads/Kb/Million (RPKM) normalized data] in different tissues of soybean were downloaded from soybean transcriptome atlas (Libault et al., 2010b). Microarray or high-throughput sequencing datasets for soybean in responses to cold, drought, phosphorus deficiency, and symbiotic bacteria, were also retrieved from previously published data (Libault et al., 2010a; Le et al., 2012; Maruyama et al., 2012; Chen et al., 2016; Zeng et al., 2016b).

### Plant Growth and Treatments

Soybean seeds (*Glycine max* var. Williams 82) were soaked in sterilized water for 4 h, and then germinated at room temperature in the dark between two layers of filter paper moistened with sterilized water (Wang et al., 2015). After 4 days, seedlings were grown hydroponically in half-strength modified Hoagland nutrient solution, the pH of the nutrient solution was adjusted to 5.6, and the nutrient solution was changed every 2 days. The seedlings were grown in a growth chamber under controlled conditions (photoperiod 16-h-light/8-h-dark at 26/22°C, light intensity 150 μmol m^-2^ s^-1^). After cultivating for 8 days, soybean seedlings were used for various stress treatments. For salt stress, the roots of seedlings were immersed in nutrient solution containing a relative high concentration of NaCl (200 mM) (Zhang et al., 2008). Plant responses to high salinity stress may be obvious and significant relative to moderate salinity stress (e.g., 100 mM). For dehydration treatment, seedlings were treated with 10% polyethylene glycol (PEG) 6000; for nutrient deficiency treatment, seedlings were transferred into normal nutrient solution (control) and nutrient-deficient solution (without phosphate, iron, or zinc). Roots of the stress-treated and non-treated plants were collected at time intervals of 0, 2, 8, and 24 h. After collection, the samples were immediately frozen in liquid nitrogen and stored at -80°C until RNA extraction.

### Quantitative RT-PCR Analysis

Gene-specific primers were designed using NCBI Primer-BLAST^9^. Primer specificity was then confirmed by blasting each primer sequence against the soybean genome. Total RNA was extracted from soybean tissues using RNApure Plant Kit (with DNase I) (CoWin Biotech, Beijing, China) and digested with DNaseI to eliminate genomic DNA contamination according to the manufacturer’s instruction. cDNA was synthesized from 1.0 μg total RNA by SuperRT Reverse Transcriptase (CoWin Biotech, Beijing, China) using oligo(dT) primers in a 20 μL reaction system. Quantitative RT-PCR (qRT-PCR) was performed on a real-time PCR system (CFX96 system, Bio-Rad) as described previously (Zeng et al., 2016a). Amplifications were run in triplicate together with controls that contained no template and no reverse transcription for each of the examined gene. Relative expression levels were normalized to that of an internal control *GmACTIN11* (*Gm18g290800*). The gene-specific primers are listed in Supplemental Table S11.

### Expression, Purification and Gel Shift Assay of Recombinant Proteins

Gene-specific primers were used to clone the coding sequences of the genes *GmCML1*, *GmCML13*, *GmCML39*, and *GmCML95* by PCR from cDNA of soybean seedlings. *AtCaM7* were cloned from Arabidopsis cDNA. The gene-specific primers are listed in Supplemental Table S11. Recombinant proteins were expressed and purified according to (Routray et al., 2013). cDNAs were subcloned into the multiple cloning sites of the pET28a expression vector (Novagen) and confirmed by DNA sequencing. Recombinant proteins were expressed in *Escherichia coli* BL21(DE3) pLysS (Stratagene). Bacterial cultures were grown under antibiotic selection in Luria-Bertani medium with agitation at 37°C to an OD600 of 0.4–0.5 and protein expression was induced over the course of 3 h by the addition of 0.5 mM isopropyl-β-D-thiogalactopyranoside (IPTG). His-tagged recombinant proteins were purified using Ni-NTA agarose affinity beads (Qiagen) as described by the manufacturer. The electrophoresis mobility-shift assay was performed as described (Garrigos et al., 1991) using 1–2 μg of denatured protein supplemented with either 1.0 mM CaCl_2_, 1.0 mM EGTA, or 1.0 mM MgCl_2_. Each sample was electrophoresed on a 12% SDS–PAGE containing either 1.0 mM CaCl_2_, 1.0 mM EGTA, or 1.0 mM MgCl_2_, respectively.

## Results

### Identification of EF-Hand Proteins in Soybean Genome

**FIGURE 1:**
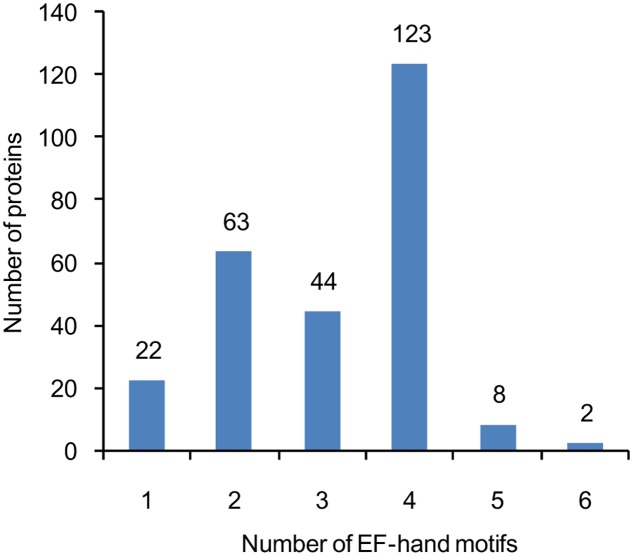
**The number of genes encoding proteins containing 1, 2, 3, 4, 5, or 6 EF-hand motifs.** The number of EF-hand motifs in each protein was predicted using InterPro database (http://www.ebi.ac.uk/interpro/).

A total of at least 262 putative EF-hand-containing proteins were found in soybean genome by using the methods described in the “Materials and Methods” section. These proteins were checked for the presence of EF-hands by using InterPro database (Hunter et al., 2009) (Supplemental Table S1). The number of EF-hand motifs in each protein varied from one to six; among these EF-hand proteins, nearly half (47%) contained four EF-hands, and 24% contained two EF-hands (**Figure 1**).

### Chromosomal Distribution of EF-Hand Proteins

**FIGURE 2:**
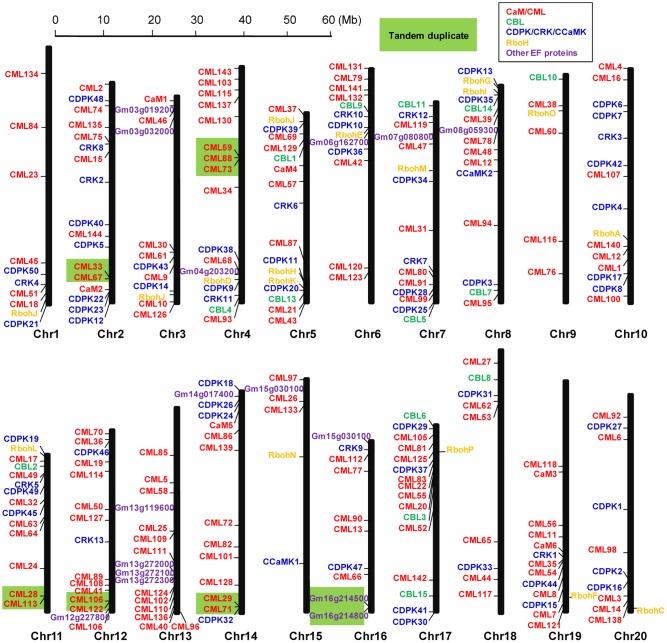
**Graphical representation of locations for putative EF-hand protein-encoding genes on each soybean chromosome.** CaM/CMLs are marked in red, CBLs are marked in green, CDPK/CRK/CCaMKs are marked in blue, respiratory burst oxidase homologs (Rbohs) are marked in orange, and the other unclassified EF-hand proteins (UEPs) are marked in purple. Genes within a light green box are putatively tandemly duplicated.

The 262 EF-hand proteins identified in this study were distributed on all of the 20 soybean chromosomes. However, the number of EF-hand proteins on each chromosome appeared to be uneven, ranging from 6 to 17 (**Figure 2**). For example, chromosome 2, 5, and 17 contained the highest number of 17 EF-hand proteins, whereas only 6 EF-hand proteins were found on chromosome 9 and 15. Tandem duplication is a factor contributing to the evolution of many gene families (Cannon et al., 2004). Genes located within 100 kb from each other and separated by five or less genes were marked with a light green box to indicate possible tandem duplications. There were 13 possible tandem duplicated sites among these EF-hand proteins (**Figure 2**).

### Classification and Phylogenetic Analysis of EF-Hand Proteins

**FIGURE 3:**
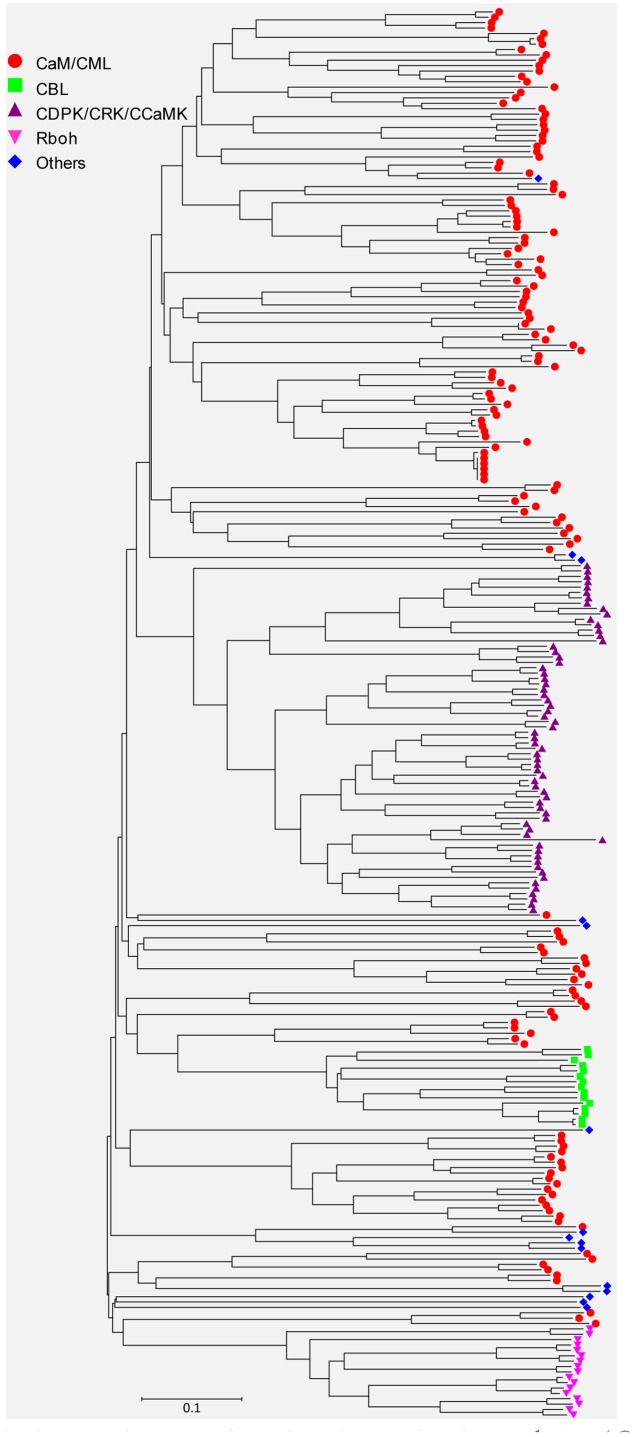
**Unrooted phylogenetic tree of EF-hand proteins in soybean (*Glycine max*).** The alignment for the phylogenetic tree was performed with ClustalW using full-length amino acid sequences. The phylogenetic tree was created with the MEGA6 software and the neighbor-joining method with 1,000 bootstrap replications. All the 150 CaM/CML proteins are marked with red circles, 15 CBL proteins are marked with green squares, 65 CDPK/CRK/CCaMK proteins are marked with purple triangles, 17 Rboh proteins are marked with pink inverted triangles, and 15 UEPs are marked with blue diamonds. The bar indicates the relative divergence of the sequences examined.

**FIGURE 4:**
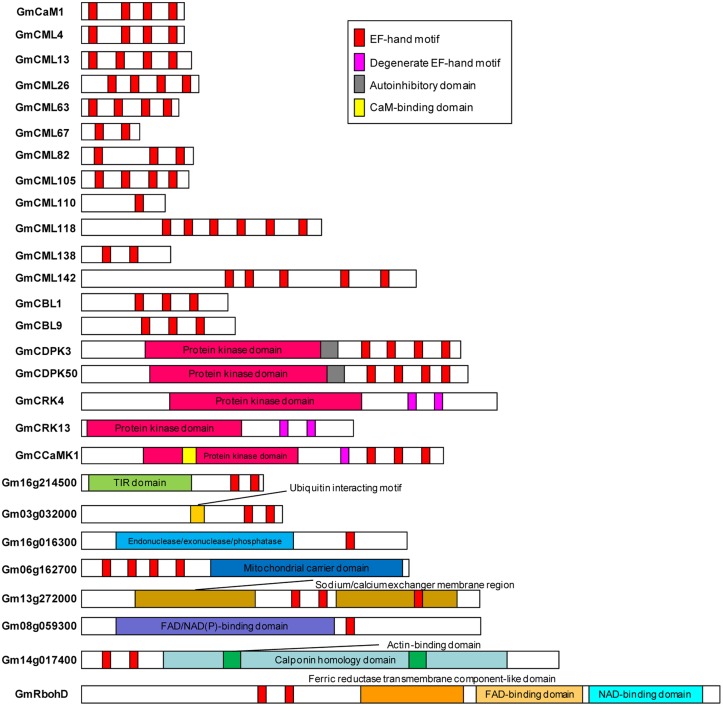
**Schematic diagrams of representative soybean EF-hand proteins.** EF-hands and other domains were predicted using InterPro database (http://www.ebi.ac.uk/interpro/).

Sequences of all the identified EF-hand proteins were aligned using ClustalW and a phylogenetic tree was constructed using the neighbor-joining method. The phylogenetic tree of the overall 262 EF-hand proteins was shown in **Figure 3**. According to the presence of predicted functional domains other than EF-hands, and their similarity to classical Ca^2+^ sensors, such as CaM/CML, CBL, and CDPK, these proteins were classified into five groups, CaM/CMLs, CBLs, CDPK/CRK/CCaMKs, Rbohs, and UEPs (Supplemental Tables S2–S7 and **Figure 3**). The schematic diagrams of representative EF-hand proteins of various groups containing different number of EF-hands were shown in **Figure 4**. As can be seen, the CaMs, CMLs, and CBLs have no functional domain other than EF-hands, while the other proteins have additional domains, such as protein kinase domain, TIR domain, mitochondrial carrier domain, actin-binding domain, and FAD-binding domain (**Figure 4**).

### CaM and CML Proteins in Soybean

**FIGURE 5:**
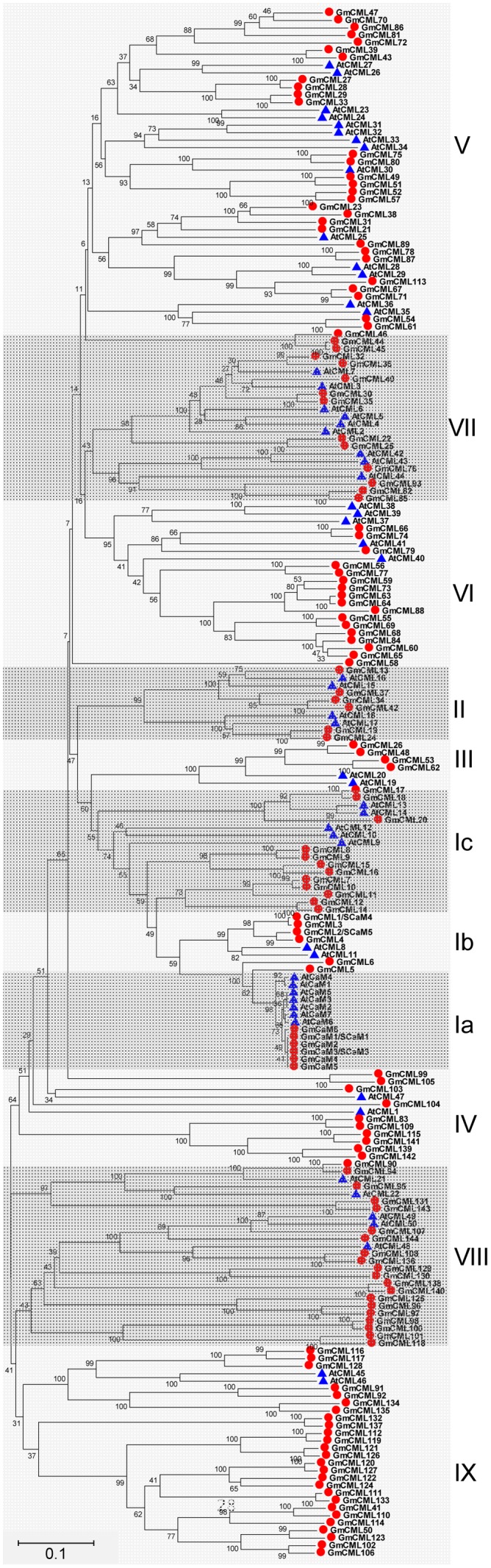
**Unrooted phylogenetic tree of the CaM and CML proteins from soybean (*G. max*) and *Arabidopsis thaliana*.** The alignment for the phylogenetic tree was performed with ClustalW using full-length amino acid sequences. The phylogenetic tree was created with the MEGA6 software and the neighbor-joining method with 1,000 bootstrap replications. All the 150 CaM/CML proteins from soybean are marked with red circles, while 57 CaM/CML proteins from Arabidopsis are marked with blue triangles. Roman numerals designate the subfamilies. The bar indicates the relative divergence of the sequences examined and bootstrap values are displayed next to the branch.

Based on the extremely high amino acid identity to *Arabidopsis thaliana* CaM2 (AtCaM2) (98.0–98.7%), six genes were found to encode two isoforms of CaMs in soybean (CaM1/2/3/4/5, CaM6), both are 149 amino acid (AA) in length. In addition, there were at least 144 genes encoding for CML proteins; these CMLs share at least 12.8% overall amino acid identity with AtCaM2, and have no other known functional domains except the EF-hands (Supplemental Table S2). The length of soybean CMLs ranged from 80 to 501 AA. Half of these CMLs (72/144) have four EF-hands, and only two CMLs (CML101 and CML118) have six EF-hands. CML1 and CML2 have previously been known as SCaM4 and SCaM5, respectively (Lee et al., 1995). However, because the encoded proteins share only 79% amino acid identity with AtCaM2, they are likely to have distinct functions from the conserved CaMs. Protein sequences of CaM/CMLs from soybean and Arabidopsis were retrieved for phylogenetic analysis, and the results showed that these CaM/CMLs can be classified into nine subgroups, with each subgroup containing different number of CaM/CML proteins (**Figure 5**).

### CBL Proteins in Soybean

**FIGURE 6:**
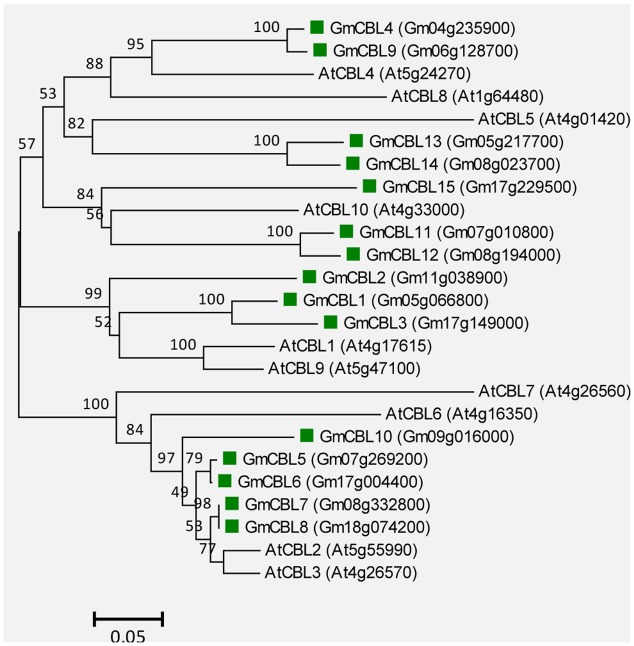
**Unrooted phylogenetic tree of the total CBL proteins from soybean (*G. max*) and *A. thaliana*.** The alignment for the phylogenetic tree was performed with ClustalW using full-length amino acid sequences. The phylogenetic tree was created with the MEGA6 software and the neighbor-joining method with 1,000 bootstrap replications. The 15 CBLs from soybean are marked with green squares, while the 10 CBLs from Arabidopsis are not marked. Locus name of each gene is shown in brackets. The bar indicates the relative divergence of the sequences examined and bootstrap values are displayed next to the branch.

A total of 15 genes encoding putative CBLs were found in soybean genome, and they showed high sequence identity with AtCBL1 (48.8–77.5%). These genes were sequentially named *GmCBL1*-*GmCBL15*, based on their amino acid identity to AtCBL1 (Supplemental Table S3). Similar to their counterparts in other plants, GmCBLs are relatively small proteins with lengths ranging from 212 to 265 AA, and all proteins contain three putative EF-hands (Supplemental Table S3). Phylogenetic analysis indicated that GmCBLs are closely related to the 10 AtCBLs (**Figure 6**).

### CDPK, CRK, and CCaMK Proteins in Soybean

**FIGURE 7:**
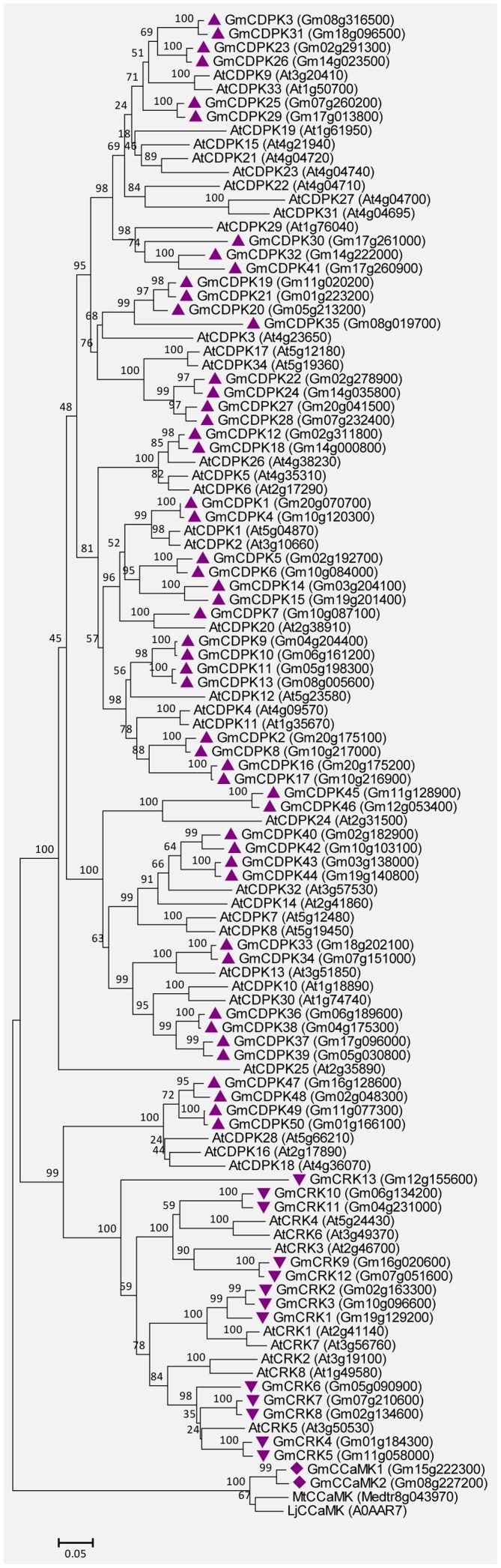
**Unrooted phylogenetic tree of the GmCDPK, GmCRK, and GmCCaMK proteins.** The alignment for the phylogenetic tree was performed with ClustalW using full-length amino acid sequences. The phylogenetic tree was created with the MEGA6 software and the neighbor-joining method with 1,000 bootstrap replications. All the 50 GmCDPKs are marked with triangles, 13 GmCRKs are marked with inverted triangles, and 2 GmCCaMKs are marked with diamonds, while proteins from Arabidopsis (34 CDPKs and 8 CRKs), and CCaMKs from *Medicago truncatula* (MtCCaMK) or *Lotus japonicus* (LjCCaMK) are not marked. Locus name of each gene is shown in brackets. The bar indicates the relative divergence of the sequences examined and bootstrap values are displayed next to the branch.

The soybean genome contains 50 CDPKs, 2 CCaMKs, and 13 CRKs. The detailed characteristics are shown in Supplemental Tables S4, S5. Most GmCDPKs contain four predicted EF-hands except for GmCDPK16, GmCDPK17, and GmCDPK35, which contain five, five, and one EF-hands, respectively (Supplemental Table S4). GmCDPK1, GmCDPK2, and GmCDPK3 have previously been defined as GmCDPKα, GmCDPKβ, and GmCDPKγ, respectively (Lee et al., 1998). Both the GmCCaMK1 and GmCCaMK2 contain three predicted EF-hands, and their amino acid identities to MtCCaMK are very high (87 and 88%). The CRKs possess the CaM-like domain with poorly conserved EF-hands (Hrabak et al., 2003). All GmCRKs were predicted to contain two degenerated EF-hands (Supplemental Table S5). In addition, 80% (40/50) of GmCDPKs and 77% (9/13) of GmCRKs have predicted myristoylation motifs at their N-termini (Supplemental Tables S4, S5), a feature observed in their orthologs from Arabidopsis (Hrabak et al., 2003). Myristoylation was reported to be an important mechanism for the membrane attachment of plant CDPKs (Martín and Busconi, 2000; Rutschmann et al., 2002). **Figure 7** shows the unrooted phylogenetic tree for CDPKs/CRKs from soybean and Arabidopsis, and also CCaMKs from soybean, *Medicago truncatula* and *Lotus japonicus*, based on the comparisons of their amino acid sequences. These GmCDPK/CRK/CCaMK proteins are closely related to their orthologs in Arabidopsis and other plants.

### Rboh Proteins in Soybean

**FIGURE 8:**
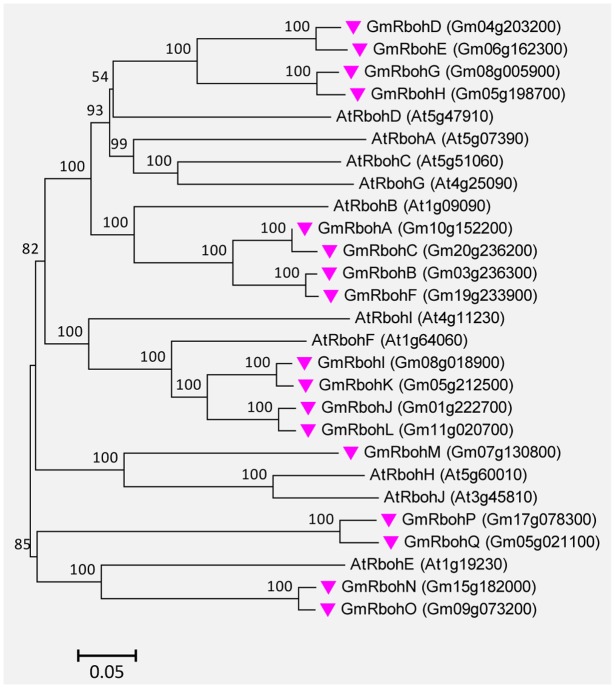
**Unrooted phylogenetic tree of the Rboh proteins from soybean (*G. max*) and *A. thaliana*.** The alignment for the phylogenetic tree was performed with ClustalW using full-length amino acid sequences. The phylogenetic tree was created with the MEGA6 software and the neighbor-joining method with 1,000 bootstrap replications. Seventeen GmRbohs are marked with pink inverted triangles, while 10 AtRboh proteins are not marked. Locus name of each gene is shown in brackets. The bar indicates the relative divergence of the sequences examined and bootstrap values are displayed next to the branch.

A total of 17 Rboh genes were found in the soybean genome. They were successively named GmRbohA-Q based on their homology to AtRbohD (Supplemental Table S6). The GmRboh proteins are 820–941 AA long and share 42–67% identity to AtRbohD. The intron numbers contained in GmRboh genes vary from 10 to 13 (Supplemental Table S6). Similar to their counterparts in Arabidopsis and other plants, all GmRboh proteins contain two putative EF-hand motifs. In addition, the evolutionary relationships between GmRbohs and 10 AtRboh members are closely related (**Figure 8**).

In addition, there are genes in soybean genome encoding 15 EF-hand proteins which are not belonging to CaM/CMLs, CBLs, CDPK/CRK/CCaMKs, and Rbohs, and they are named UEPs, (Supplemental Table S7). For example, *UEP8* (*Gm13g272000*), *UEP9* (*Gm13g272100*), and *UEP10* (*Gm13g272300*) are tandemly duplicated genes encoding EF-hand proteins with putative sodium/calcium exchanger function (**Figures 2**, **4**).

### *cis*-Acting Regulatory Elements in the Promoters of EF-Hand Genes

It is well known that transcription of a gene in plants is usually altered when an appropriate transcription factor recognizes and binds to a specific DNA motif (*cis*-element). Here we surveyed the presence of eight classes of *cis*-elements (Supplemental Table S8) related to hormonal signal and/or stress responses, in the -1500 bp promoter regions upstream to the predicted transcription start sites of these EF-hand genes (Williams et al., 1992; Abe et al., 1997; Ulmasov et al., 1997; Fujimoto et al., 2000; Sakuma et al., 2002; Yang and Poovaiah, 2002; Osakabe et al., 2014; Ruan et al., 2015). Of these 262 EF-hand genes, 39 contain dehydration- and cold-responsive element DRE/CRT, 78 contain ABA-responsive element ABRE, 112 contain auxin-responsive element AuxRE, 17 contain ethylene-responsive element GCC-box, 45 contain environmental response-related element G-box, 81 contain signal response-related element CG-box, 28 contain drought and ABA signaling-related element MYB2-BS, and 66 contain phosphate starvation response-related element P1BS (Supplemental Table S9). However, 32 genes did not contain any of these elements (Supplemental Table S9). Interestingly, more than one type of these *cis*-elements exist in the promoters of 142 EF-hand genes. For example, there are five types of *cis*-elements in the promoters of *CML15*, *CML40*, *RbohB*, *RbohE*, *UEP3 (Gm06g162700)*, and *UEP12 (Gm15g030100)* (Supplemental Table S9). The enrichment of hormone-/stress-responsive *cis*-elements in the promoters of these EF-hand genes suggests that they are likely to be involved in plant responses to various hormone signals and stresses.

### Tissue-Specific Expression of EF-Hand Genes

**FIGURE 9:**
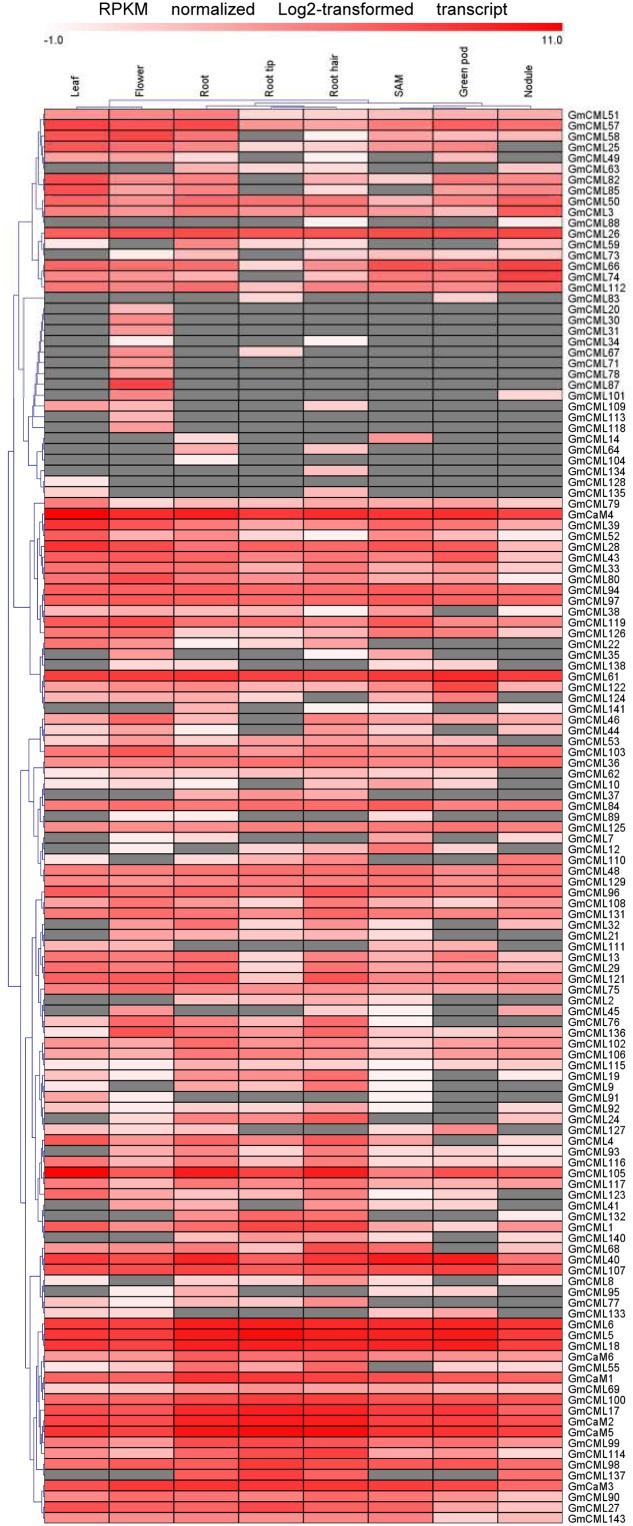
**Heat map representation for tissue-specific expression patterns of the predicted *GmCaM/CML* genes according to Illumina transcriptome data.** The Reads/Kb/Million (RPKM) normalized log2 transformed counts were visualized in the heat map. The red colors indicate expression intensity, gray color indicates no expression.

**FIGURE 10:**
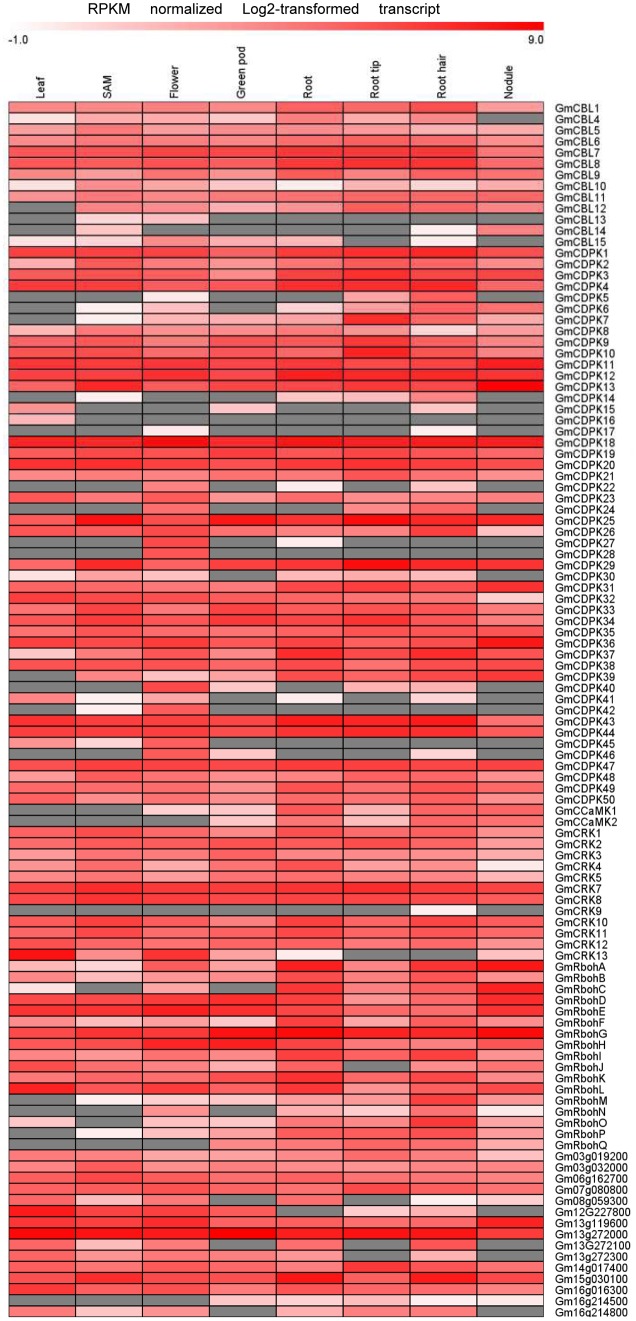
**Heat map representation for tissue-specific expression patterns of predicted EF-hand protein-coding genes with the exception of *GmCaM/CML*s according to Illumina transcriptome data.** The RPKM normalized log2 transformed counts were visualized in the heat map. The red colors indicate expression intensity, gray color indicates no expression detected.

Transcriptome data derived from Illumina sequencing were used to assess the expression patterns of EF-hand genes in different tissues of soybean. The transcriptome atlas provided expression data for 55,616 putative soybean genes in eight types of tissues and organs, including root tips, roots, root hairs, nodules, leaves, shoot apical meristems, flowers and green pods (Libault et al., 2010b). The expression data of almost all EF-hand genes can be found in the transcriptome atlas, with the exception of five genes (*CML56*, *CML65*, *CML120*, *CML144*, and *CRK6*) (**Figures 9**, **10**). However, 17 genes were found to have no expression in any of these tissues; these genes are *CML11*, *CML15*, *CML16*, *CML23*, *CML42*, *CML47*, *CML54*, *CML60*, *CML70*, *CML72*, *CML81*, *CML86*, *CML130*, *CML139*, *CML142*, *CBL2*, and *CBL3*. These genes could be pseudogenes or could be expressed only under specific developmental stages or environmental conditions which were not met in these studies. As shown in the heat maps, some genes were ubiquitous in various tissues, while some genes are tissue-specific (**Figures 9**, **10**). For example, *CML6*, *CML18*, *CML26*, *CML48*, *CML61*, *CBL6*, *CBL11*, *CDPK18*, *CDPK19*, and *CDPK20* were constitutive expressed in various tissues; *CML20*, *CML30*, *CML71*, *CML78*, *CML87*, *CML113*, *CML118*, and *CDPK28* were specifically expressed in flower tissue; *CaM2*, *CML2*, *CML9*, *CML19*, *CML24*, *CML55*, *CML77*, *CML137*, *CDPK6*, *CDPK14*, *CCaMK1*, *CCaMK2*, and *RbohN* were preferentially expressed in roots/root tips/root hairs (**Figures 9**, **10**).

### Transcriptional Responses of EF-Hand Genes to Stresses

**FIGURE 11:**
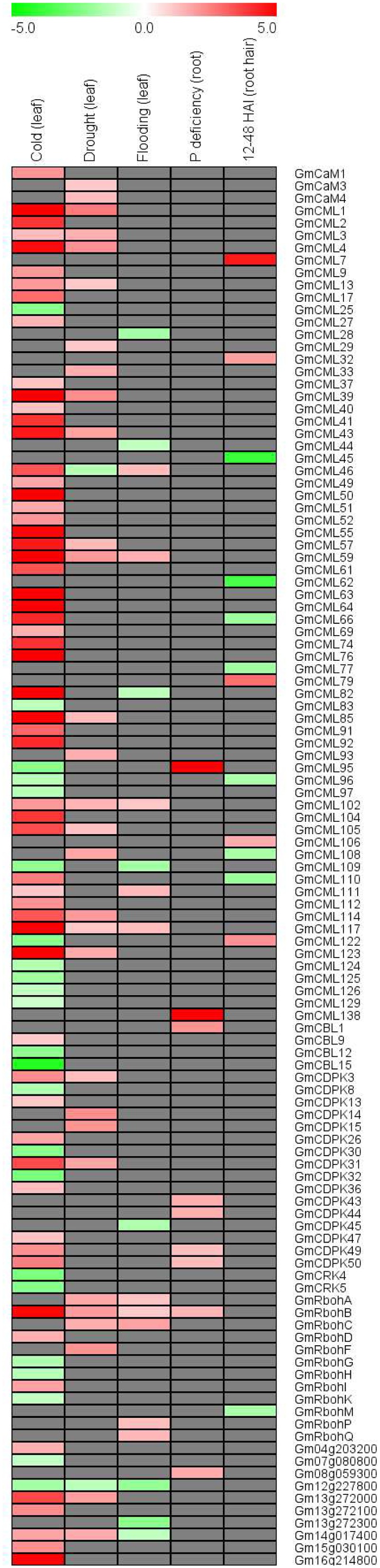
**Heatmap representation for expression profiles of EF-hand protein-encoding genes in response to cold, drought, flooding, phosphorus deficiency, and *Bradyrhizobium japonicum* inoculation (24–48 HAI).** The intensities of the color represent the relative magnitude of fold changes in log2 values according to microarray or high-throughput sequencing data. Red color indicates induction, green color indicates repression, gray color means there is no significant expressional change.

In order to investigate whether the EF-hand genes are responsive to environmental stresses, we retrieved the published soybean microarray and/or deep sequencing data reflecting soybean responses to various stresses including cold, drought, flooding, phosphate deficiency, and *Bradyrhizobium japonicum* inoculation (Libault et al., 2010a; Le et al., 2012; Maruyama et al., 2012; Chen et al., 2016; Zeng et al., 2016b). Of all the EF-hand genes, 113 (43.1%) genes were shown to be differentially expressed under one or multiple stresses (**Figure 11**). For example, *CML59*, *CML102*, *CML117*, and *RbohB* were induced by cold, drought, and flooding; *CML109* and *UEP6* (*Gm12g227800*) were repressed by cold and flooding; *CML82* and UEP11 (*Gm14g017400*) were induced by cold but repressed by flooding; *CML28*, *CDPK45*, and *UEP10* (*Gm13g272300*) were only responsive to cold stress.

**FIGURE 12:**
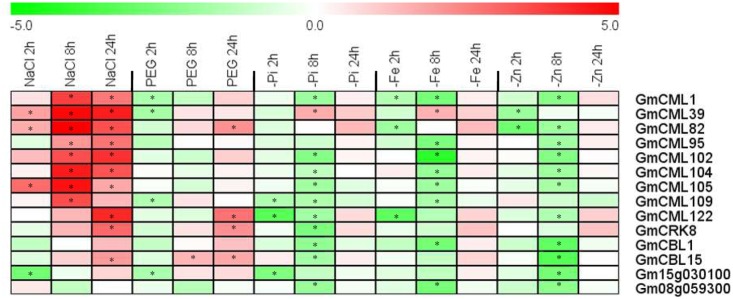
**Heat map representation for expression profiles of some selected EF-hand genes in response to stresses.** The expression of EF-hand genes were analyzed by qRT-PCR in roots of 12-day-old seedling, after treatment with 200 mM NaCl (salt), 10% PEG 6000, phosphate deficiency (-Pi), iron deficiency (-Fe), and zinc deficiency (-Zn) for 2, 8, and 24 h. The intensities of the color represent the relative magnitude of fold changes (treatment/control) in log2 values of three technical replicates. The asterisks indicate an absolute fold change ≥ 2 and *P*-value < 0.05 by Student’s *t*-test. Red color indicates induction, green color indicates repression.

In addition, we also selected 14 EF-hand genes to confirm their transcriptional responses to environmental stresses including salt, dehydration, phosphate deficiency, iron deficiency, and zinc deficiency by qRT-PCR (**Figure 12**). Nearly all of these genes were responsive to diverse stresses. Most of them were induced by salt stress, but were repressed by various nutrient deficiencies, and eight genes of them were responsive to PEG treatment (dehydration), four were induced (*CML82*, *CML122*, *CRK8*, and *CBL15*) and four [*CML1*, *CML39*, *CML109*, and *UEP12* (*Gm15g030100*)] were repressed (**Figure 12**).

### Confirmation of the Ca^2+^-Binding Ability of EF-Hand Proteins

**FIGURE 13:**
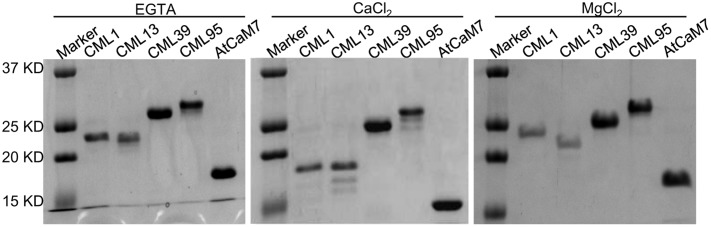
**Effect of Ca^2+^ on the electrophoretic mobility of GmCML1, GmCML13, GmCML39, and GmCML95 proteins.** Samples (1–2 μg) of purified, recombinant AtCaM7 (positive control), GmCML1, GmCML13, GmCML39, and GmCML95 containing 1.0 mM CaCl2, 1.0 mM EGTA, or 1.0 mM MgCl_2_ were separated on SDS–PAGE gels supplemented with 1.0 mM CaCl_2_, 1.0 mM EGTA, or 1.0 mM MgCl_2_, respectively, and stained with Coomassie Brilliant Blue. The positions of the molecular weight markers (kDa) are indicated on the left.

Based on the gene expression analysis, we further examined four representative soybean CMLs (CML1, CML13, CML39, and CML95) to confirm their Ca^2+^-binding ability by using SDS–PAGE mobility-shift assay. The accelerated electrophoretic migration of CaM/CMLs in the presence of Ca^2+^ is a well-documented phenomenon (Garrigos et al., 1991; Vanderbeld and Snedden, 2007). As shown in **Figure 13**, like the conserved CaM, the CML1, CML13, and CML39 exhibit the characteristic Ca^2+^ shift, and CML95 exhibit a small Ca^2+^ shift. Interestingly, a detectable increased shift in the mobility of CML39 was observed in the presence of MgCl_2_. We used IPD3, a non-Ca^2+^-binding protein (Routray et al., 2013) for negative control, and no shift was observed in the presence of CaCl_2_ or MgCl_2_ (Supplemental Figure S1). Mg^2+^ binding to some particular EF-hand proteins was reported for affecting EF-hand protein stability and functions (Wingard et al., 2005; Gifford et al., 2007; Vanderbeld and Snedden, 2007).

## Discussion

Ca^2+^ mediated signaling is involved in various developmental processes and responses to a variety of abiotic and biotic stresses. The Ca^2+^ binding EF-hand motif is highly conserved in most of the Ca^2+^ sensors. CaMs/CMLs, CBLs, and CDPKs are the major subfamilies of EF-hand Ca^2+^ sensors (Weinl and Kudla, 2009; DeFalco et al., 2010; Poovaiah et al., 2013). Although subfamilies of Ca^2+^ sensors (e.g., CaM/CML, CBL, and CDPK) have been analyzed in model plants and some major crops, no detailed information about these Ca^2+^ sensors in soybean is systemically reported so far (Hrabak et al., 2003; McCormack and Braam, 2003; Kolukisaoglu et al., 2004; Asano et al., 2005; Boonburapong and Buaboocha, 2007; Chen et al., 2013; Kong et al., 2013; Zhao et al., 2013; Kleist et al., 2014; Meena et al., 2015; Munir et al., 2016). Using domain prediction programs and BLAST searches against known EF-hand proteins (e.g., AtCaM2, AtCBL1, AtCDPK1, AtCRK1, and AtRbohD) at the genomic level in soybean, at least 262 genes encoding proteins with varying number of EF-hand motifs were identified (**Figures 1–3**). The EF-hand genes in soybean include 6 *CaM* genes, 144 *CML* genes, 15 *CBL* genes, 50 *CDPK* genes, 13 *CRK* genes, 2 *CCaMK* genes, 17 *Rboh* genes, and 15 UEP genes (Supplemental Tables S1–S7). EF-hand motifs tend to occur in pairs, which can increase stability as well as affinity of binding between the EF-hand proteins and Ca^2+^ (Gifford et al., 2007). Consistent with the EF-hand family members in other plants, the majority of the soybean EF-hand proteins (71.8%) have pairs of EF-hand motifs (two, four, or six) (**Figure 1**) (Day et al., 2002; McCormack and Braam, 2003; Boonburapong and Buaboocha, 2007; Munir et al., 2016).

The number of total EF-hand proteins in Arabidopsis and rice plants were previously found to be 250 and 243, respectively (Day et al., 2002; Boonburapong and Buaboocha, 2007). The number of EF-hand proteins in soybean is similar to that found in Arabidopsis and rice. However, the number of CMLs in soybean (144) is much more than that in Arabidopsis (50), rice (32), and tomato (52) (McCormack and Braam, 2003; Boonburapong and Buaboocha, 2007; Munir et al., 2016). In addition, the number of CBLs, CDPKs, CRKs, and Rbohs in soybean is at least 1.5-folds as much as that in Arabidopsis and soybean (Supplemental Table S10). The large size of the CML/CBL/CDPK/CRK/Rboh gene families in soybean could be attributed to the whole-genome duplication events occurred approximately 59 and 13 million years ago (Schmutz et al., 2010). However, the EF-hand proteins other than CaMs/CMLs/CBLs/CDPKs/CRKs/CCaMKs in soybean are much less than that in Arabidopsis and rice plants (Supplemental Table S10). This may be caused by reasons that some loci encoding putative EF-hand proteins are not annotated in the current version of soybean genome (version 2.0), or the composition of different subfamilies of EF-hand Ca^2+^ sensor may be a little different between legume and non-legume plants.

The expression patterns of EF-hand genes in different tissues revealed that these genes were diversely expressed in various tissues of soybean (**Figures 9**, **10**). Ca^2+^ sensor subfamilies, such as CaM/CMLs and CDPKs also show tissue and developmentally specific expression patterns (McCormack et al., 2005; Kong et al., 2013; Munir et al., 2016). It is clear that Ca^2+^ signaling is highly complex and plays an important role during plant growth and development. For example, Ca^2+^/CaM signaling is critical for brassinosteroid biosynthesis and plant growth by regulating the function of DWARF1 (Du and Poovaiah, 2005); Arabidopsis CML42 regulates trichome branching by interacting with KIC (kinesin interacting Ca^2+^-binding protein) (Dobney et al., 2009); CBL-interacting protein kinase CIPK6 is involved in root development (Tripathi et al., 2009); CDPK28 functions as a regulatory component controlling stem elongation and vascular development (Matschi et al., 2013); Arabidopsis RbohC regulates root cell expansion through the activation of Ca^2+^ channels (Foreman et al., 2003). Ca^2+^ signaling-components, such as DMI1 (does not make infections 1), Ca^2+^-dependent ATPase MCA8, the cyclic nucleotide-gated channels (CNGCs) and CCaMK are required for the establishment of root symbiosis in legumes (Oldroyd, 2013; Poovaiah et al., 2013; Charpentier et al., 2016). Interestingly, some EF-protein-coding genes were preferentially expressed in root nodule of soybean, such as *CML45*, *CML63*, *CML73*, *CML110*, *CDPK6*, CDPK14, *CCaMK1*, and *CCaMK2* (**Figures 9**, **10**). Notably, *CML45* and *CML110* were responsive to the inoculation of rhizobia (*B. japonicum*) (**Figure 11**). Whether these genes are possibly associated with symbiosis establishment or nodule development should deserve further researches.

A variety of stimuli including hormonal signals, abiotic and biotic stresses, regulate the expression of diverse subfamilies of EF-hand-containing proteins (McCormack et al., 2005; Boudsocq and Sheen, 2013; Chen et al., 2013; Kong et al., 2013; Zhao et al., 2013; Meena et al., 2015; Mohanta et al., 2015; Munir et al., 2016). Nowadays, a lot of EF-hand proteins have been documented to mediate plant response and tolerance to various environmental stresses (Weinl and Kudla, 2009; Xi et al., 2012; Bender and Snedden, 2013; Boudsocq and Sheen, 2013; Zhang et al., 2014; Zeng et al., 2015). For example, Arabidopsis CaM3 is involved in heat shock signal transduction because *cam3* mutant was more sensitive to heat stress (Zhang et al., 2009); Arabidopsis *CML9* knockout mutant plants express more tolerance to salinity and drought (Magnan et al., 2008); overexpression of *GmCaM4* confers soybean enhanced resistances to pathogens and salt stress (Rao et al., 2014); overexpression of rice *CML4* enhances drought tolerance in transgenic rice (Yin et al., 2015); Arabidopsis *cml42* mutant plants are more resistant to herbivory than the wild type plants (Vadassery et al., 2012); Arabidopsis CBL1, CBL2, CBL3, CBL4, CBL9, and CBL10 are involved in cellular ion homeostasis regulation (e.g., Na^+^, K^+^, Mg^2+^, and NO_3_^-^) (Weinl and Kudla, 2009; Tang et al., 2015); overexpression of Arabidopsis CBL5 and soybean CBL1 improves salt and drought tolerance in transgenic Arabidopsis plants (Cheong et al., 2010; Li et al., 2012); Arabidopsis CDPK8 functions in ABA-mediated stomatal regulation in response to drought by phosphorylating CATALASE3 (Zou et al., 2015); rice CPK21 positively regulates salt stress tolerance (Asano et al., 2011); overexpression of a Medicago EF-hand protein gene MtCaMP1 enhances drought and salt stress tolerance (Wang et al., 2013). In the present study, 43.1% of all the EF-hand genes were found to be differentially expressed under one or multiple stresses including cold, drought, salinity, flooding, rhizobia inoculation, and nutrient deficiencies of phosphate, iron, or zinc (**Figures 11**, **12**). It is notable that most of these genes (85%) contain at least one type of stress-related *cis*-elements in their promoters (Supplemental Table S9). In this study, we did not analyzed the physiological role of any soybean EF-hand proteins. But in the future, researches through the combination of biochemical, molecular, and genetic approaches are required to dissect the exact physiological roles of these EF-hand proteins considering the important functions of their counterparts in model plants and their responsiveness to one or multiple stresses.

## Conclusion

In this study, a total of at least 262 genes encoding proteins with varying number of EF-hand motifs were identified in soybean genome, including 6 *CaM* genes, 144 *CML* genes, 15 *CBL* genes, 50 *CDPK* genes, 13 *CRK* genes, 2 *CCaMK* genes, 17 *Rboh* genes, and 15 *UEP* genes. Most of these genes (87.8%) contain one or multiple hormonal signal and/or stress-responsive *cis*-elements in the -1500 bp promoter regions. Expression profiling revealed that these EF-hand genes were broadly expressed in different organs of soybean. Expression pattern analyses also revealed that nearly half of these genes (43.1%) could be induced or repressed under various environmental or nutritional stresses, indicating their potential role in stress responses. Further in-depth functional characterization will enhance our understanding of Ca^2+^-mediated signaling underling plant responses to environmental and nutritional stresses and facilitate the development of stress resistant crops.

## Author Contributions

HZ conceived and designed the study, and wrote the manuscript. HZ, YxZ, and XZ performed the bioinformatic analysis and the experiments. EP and YyZ participated in the data analysis. All authors read and approved the manuscript.

## Conflict of Interest Statement

The authors declare that the research was conducted in the absence of any commercial or financial relationships that could be construed as a potential conflict of interest.

## Footnotes

1 https://phytozome.jgi.doe.gov/pz/portal.html

2 http://www.ebi.ac.uk/interpro/

3 http://www.arabidopsis.org/

4 https://phytozome.jgi.doe.gov/pz/portal.html

5 https://phytozome.jgi.doe.gov/jbrowse/index.html

6 http://www.ebi.ac.uk/interpro/

7 http://mendel.imp.ac.at/myristate/SUPLpredictor.htm

8 http://floresta.eead.csic.es/rsat/

9 http://www.ncbi.nlm.nih.gov/tools/primer-blast/
