# Supplementary material for: Analysis of EF-Hand Proteins in Soybean Genome Suggests Their Potential Roles in Environmental and Nutritional Stress Signaling
Source: Front Plant Sci. 2017 May 24;8:877. doi: 10.3389/fpls.2017.00877 (PMC5443154; doi:10.3389/fpls.2017.00877)
Supplement: Supplementary file 2 [file Data_Sheet_1.DOCX]

Supplemental Figure S1. Effect of Ca^2+^ on the electrophoretic mobility of a non-Ca^2+^-binding protein IPD3. Purified recombinant IPD3 (segment of 68-265 aa) (0.5µg) was separated on SDS-PAGE gels supplemented with 1.0 mM CaCl_2_, 1.0 mM EGTA or 1.0 mM MgCl_2_, respectively and stained with Coomassie Brilliant Blue. The red arrow indicates the position of IPD3 segment. The positions of the molecular weight markers (kDa) are indicated on the left.
